# Transcriptome analysis of the salivary glands of the grain aphid, *Sitobion avenae*

**DOI:** 10.1038/s41598-017-16092-z

**Published:** 2017-11-21

**Authors:** Yong Zhang, Jia Fan, Jingrui Sun, Frédéric Francis, Julian Chen

**Affiliations:** 1 State Key Laboratory of Plant Diseases and Insect Pests, Institute of Plant Protection, Chinese Academy of Agricultural Sciences, Beijing, 100193 P.R. China; 2 Functional and Evolutionary Entomology, Gembloux Agro-Bio Tech, University of Liège, Gembloux, B-5030 Belgium

## Abstract

Aphid saliva plays important roles in aphid-host interactions, such as assisting aphid digestion, detoxification, activating or suppressing plant defenses. The grain aphid, *Sitobion avenae*, is one of the most devastating pests of cereals worldwide. In this study, we performed the transcriptome analysis of salivary glands of *S*. *avenae*. A total of 33,079 assembled unigenes were identified in the salivary glands of aphids. Of the all obtained unigenes, 15,833(47.86%) and 10,829(32.73%) unigenes showed high similarity to known proteins in Nr and Swiss-Prot databases respectively. 526 unigenes were predicted to encode secretory proteins, including some digestive and detoxifying enzymes and potential effectors. The RT-PCR and RT-qPCR results showed that all of the 15 most highly expressed putative secretory proteins specifically expressed in salivary glands. Interestingly, 11 of the 15 most highly expressed putative secretory proteins were still not matched to function-known proteins. We also detected the expression of 9 interested putative secretory proteins in aphid different tissues, including some digestive and detoxifying enzymes, effectors and Ca^2+^ binding proteins. The results showed that only glutathione-S-transferase 1 was specifically expressed in salivary glands. These findings provide a further insight into the identification of potential effectors involving in aphid-cereals interactions.

## Introduction

The salivary components of insects are thought to play crucial roles during interactions with host plants. During feeding, insects secrete saliva with wide composition into host plants for ingesting nutrients and degrading toxins^1,2^. Saliva also contains some proteins and small molecules that have been discovered to activate or suppress plant defense responses^3–6^.

Unlike leaf chewing insects such as lepidopterans, aphids (Hemiptera: Aphidoidea) have highly modified piercing-sucking mouthparts (stylets) that can penetrate between plant cells to feed phloem sap from sieve elements^7^. During probing and feeding, aphids secrete two types of saliva: gelling saliva, which solidifies into a tube-like sheath to protect the stylet from mechanical damage and chemical attack, and watery saliva, which is secreted into plant cells, intercellular spaces and phloem^8–10^. The feeding process of aphids is similar to the infection process of plant pathogens, with an interplay between aphid and host plants that follows the plant–pathogen model proposed by Jones and Dangl^11,12^. Pathogens deliver effectors into the host to modulate plant immunity using a specific secretion system^13,14^. Aphids are also thought to secrete some salivary proteins as effectors into their host plants to alter cell metabolic processes and modulate plant defense responses^15,16^.

Recently, a number of studies have focused on the function of salivary proteins during aphid–host interactions. Salivary proteins between 3 and 10 kD of the green peach aphid, *Myzus persicae*, can elicit plant defense responses in *Arabidopsis thaliana* ^17^. Salivary secretions by aphids can also prevent phloem from clogging as a result of a plant wound response to enable phloem feeding to continue^18^. The essential roles of salivary protein C002 in the successful feeding of the pea aphid, *Acyrthosiphone pisum*, on fava bean have been demonstrated using RNA interference^19^. Overexpression of *M*.*persicae* effector MpC002, potato aphid *Macrosiphum euphorbiae* effectors Me10 and Me23 in *Nicotiana benthamiana* increased aphid fecundity, suggesting their ability to suppress plant defenses, however, in *planta* expression of Mp10 and Mp42 reduced aphid fecundity and Mp10 induced obvious chlorosis response in *N*. *benthamiana*, suggested these effectors involved in the activation of plant defense response^20–22^.

For investigating the roles of saliva in aphid–plant interactions, the composition of aphid salivary proteins first needs to be identified. Actually, the transcriptome of salivary glands has been analyzed in some hemipteran species such as potato leafhopper *Empoasca fabae* ^23^, whitefly *Bemisia tabaci* ^24^, rice brown planthopper *Nilaparvata lugens* ^25^, green rice leafhopper *Nephotettix cincticeps* ^26^, tarnished plant bug *Lygus lineolaris* ^27^. Some salivary proteins with enzymatic activities were also identified in the watery saliva of *M*. *persicae* using mass spectrometry (LC-MS/MS); e.g., glucose oxidase, first identified in the chewing insect *Helicoverpa zea*, was demonstrated to be an effector that suppresses plant defense^4,28^. In a dual transcriptomic-proteomic approach, over 300 secretory salivary proteins from the salivary glands of *A*. *pisum* were identified^29^.

The grain aphid, *Sitobion avenae*, is an important agricultural pest of cereals causing serious economic losses through nutrient robbing and transmitting plant viruses (BYDV)^30,31^. In research to elucidate the composition and function of *S*. *avenae* saliva, pectinase was detected, and exogenous application of pectinase induced volatile emissions in wheat and attracted the aphid parasitoid *Aphidius avenae* ^32^. Ma *et al*. found polyphenol oxidase (PPO) in *S*. *avenae* saliva, and wheat seedlings treated with PPO had increased expression of genes related to plant defense signaling^33^. Recently, the composition of watery salivary proteins of *S*. *avenae* was studied by tandem mass spectrometry, and 12 proteins were identified^34^.

**Figure 1:**
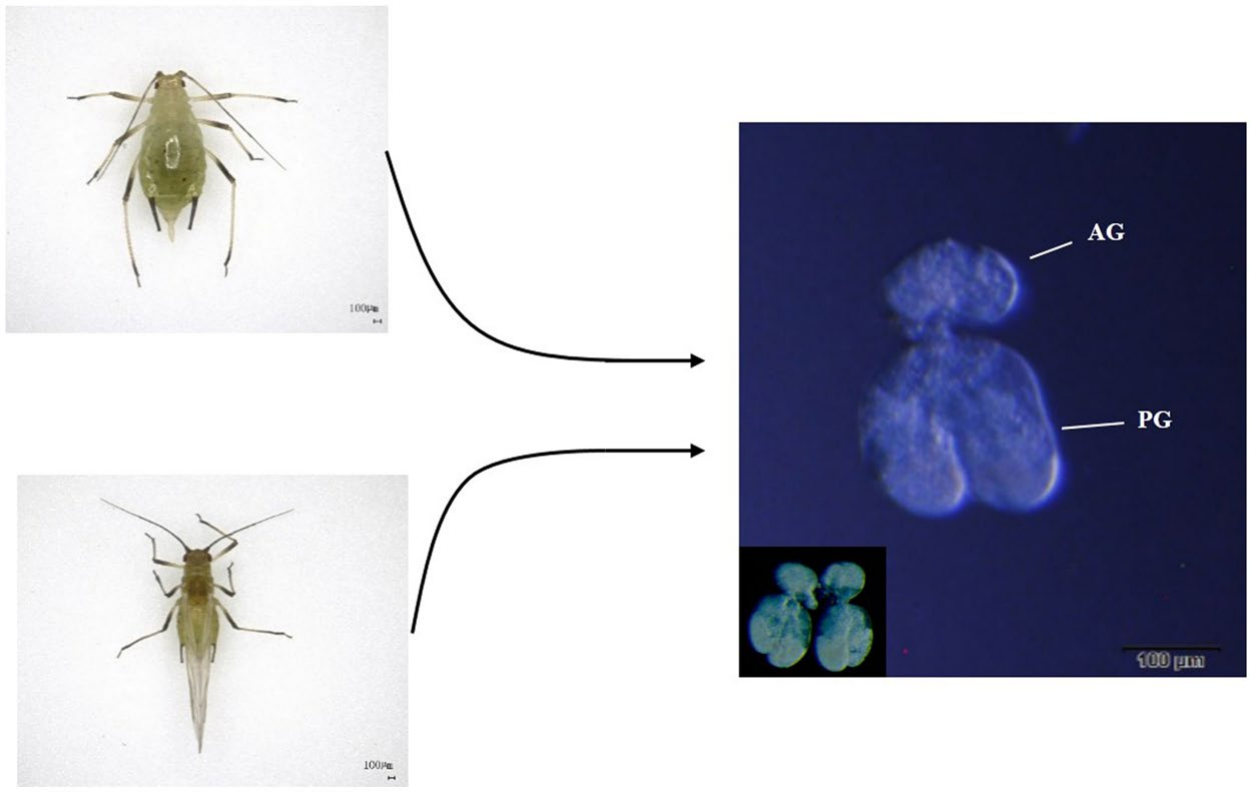
Paired salivary glands of *Sitobion avenae*, consisting of two principal glands (PG) and accessory salivary glands (AG). Photos of *S*. *avenae* were imaged by digital microscope VHX-2000C (KEYENCE, OSA, Japan) and salivary gland was scanned with stereomicroscope Olympus SZX16 (Olympus, TYO, Japan).

Aphid salivary secretory proteins were usually collected from artificial diet for further identification by proteomics, some proteins with low concentrations or only induced during interacted with host plants may not be detected. Aphid salivary glands are paired and composed of two primary glands and two accessory glands, secreted salivary proteins are mainly synthesized in the primary glands^35^. Although the function of the accessory glands has not been well studied, they might be involved in the transmission of viruses such as BYDV in *S*. *avenae* ^36^. Therefore, in this study, we directly dissected whole salivary glands from aphids (Fig. 1) and sequenced the transcriptome of the salivary glands of *S*. *avenae*. Of 33,079 unigenes identified in the whole salivary glands, 526 unigenes were predicted to encode secretory proteins, and some of their orthologs have been proved to play important roles in aphid-host interactions. These findings firstly provide insight into the identification of potential effector molecules in *S*. *avenae* saliva and further our understanding of the roles of saliva in aphid–wheat interactions.

## Results and Discussions

### Illumina sequencing and unigene assembly

**Figure 2:**
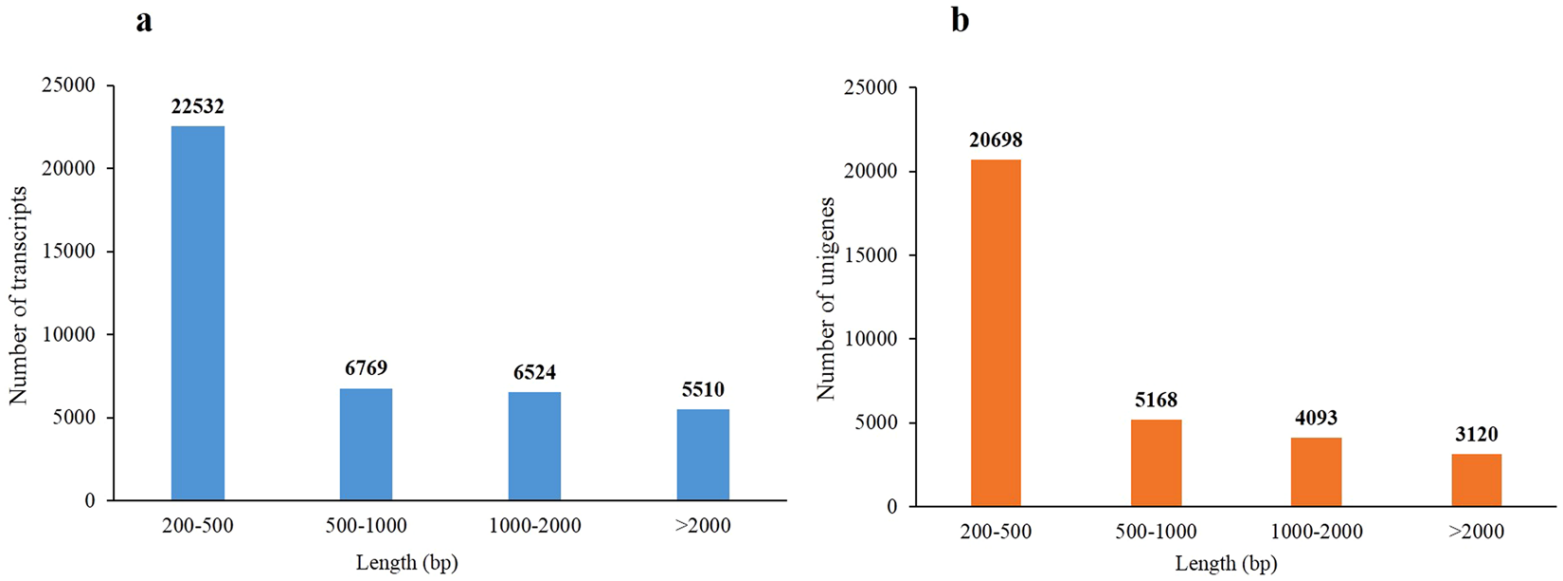
Length distribution of transcripts (**a**) and unigenes (**b**) in transcriptome assembly for *S*. *avenae* salivary glands.

In total, 58,224,912 bp and 50,854,016 bp raw reads were acquired from salivary glands of apterous and alate *S*. *avenae* adult, respectively. After removing adapters, ambiguous nucleotides and low quality sequences, 56,280,842 bp and 49,104,484 bp cleans reads remained. Subsequently, the transcriptome of *S*. *avenae* salivary glands was *de novo* assembled using the short reads assembling program-Trinity^37^, which were then clustered into 41,335 transcripts and 33,079 unigenes (Table 1). These transcripts ranged from 201 to over 14,923 bp with an average size of 941 bp. Among the transcripts, 22,532 (54.51%) were between 200 bp and 500 bp long, and 5,510 (13.33%) were over 2,000 bp. Also, among the assembled unigenes, 20,698 (62.57%) unigenes were between 200 bp and 500 bp long, and 3,120 (9.43%) were over 2,000 bp; mean length of unigenes was 711 bp (Fig. 2). All sequences of the unigenes in this study are provided in (Supplementary Table 1).

**Table 1:** The quality of *S*. *avenae* salivary glands unigene sequences and assembly.

Statistic	apterous adult	alate adult
Raw reads (bp)	58,224,912	50,854,016
Clean reads (bp)	56,280,842	49,104,484
Clean base pairs (Gb)	8.44	7.37
Error (%)	0.02	0.02
Q20 (%)	96.79	96.68
Q30 (%)	91.98	91.75
GC (%)	38.48	38.58
Unigenes	33,079

### Functional annotation of transcripts

For functional annotation, sequence similarity searches of 33,079 unigenes were run against Nr, Nt, KEGG, Swiss-Prot, PFAM, GO and KOG databases. The results showed that 15,833 (47.86%), 18,624 (56.3%), 6,772 (20.47%), 10,829 (32.73%), 10,602 (32.05%), 10,776 (32.57%) and 7,956 (24.05%) unigenes matched to known proteins in the Nr, Nt, KEGG, SwissProt, PFAM, GO and KOG databases, respectively (Table 2).

**Table 2:** Number of unigenes annotated in seven public databases.

Database	Number of unigenes	Percentage of unigenes (%)
NR	15833	47.86
NT	18624	56.3
KO	6772	20.47
Swiss-Prot	10829	32.73
PFAM	10602	32.05
GO	10776	32.57
KOG	7956	24.05
All databases	4663	14.09
At least one database	20783	62.82
Total unigenes	33079	100

**Figure 3:**
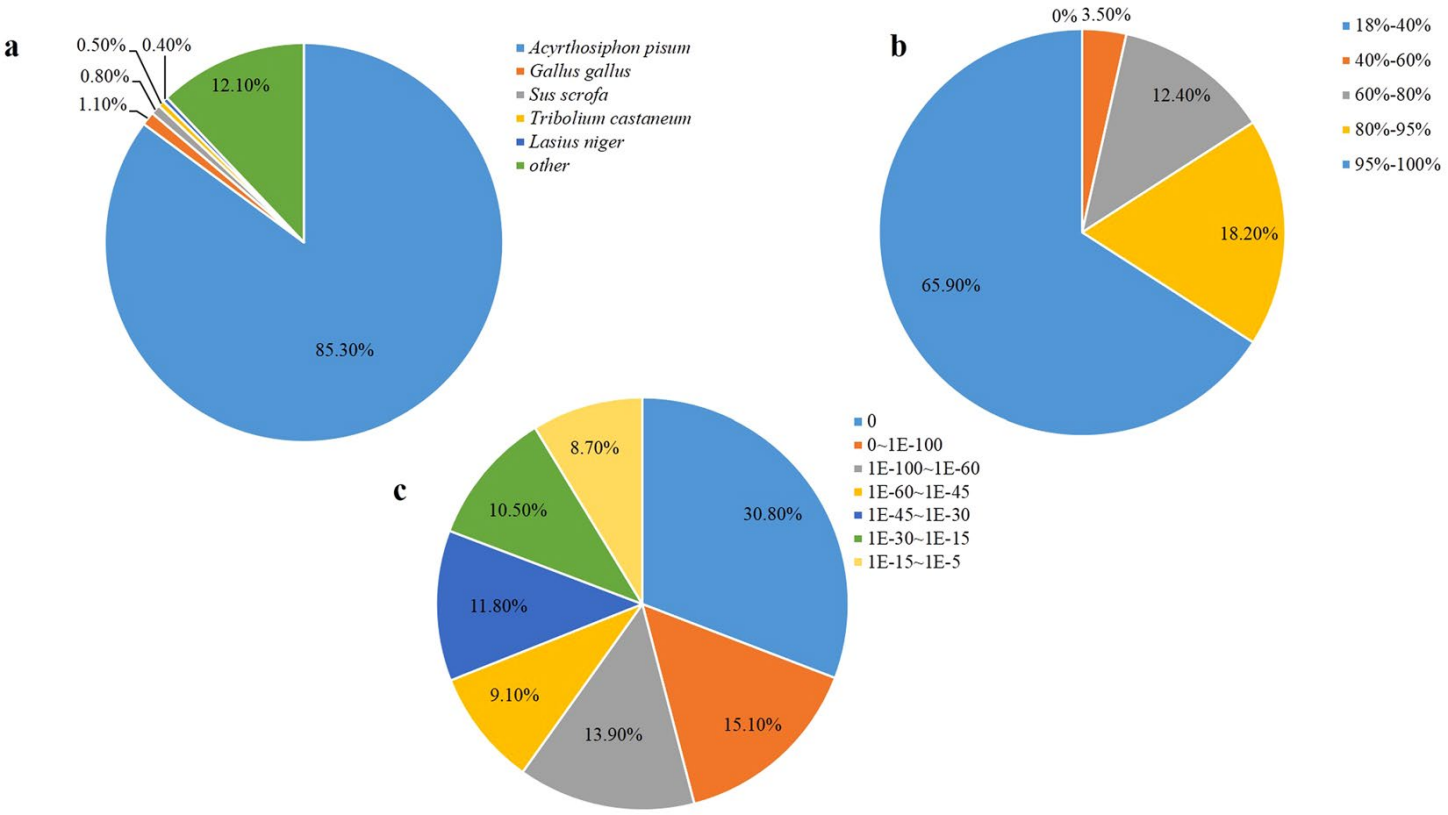
Results of similarity search of unigenes against Nr database. (**a**) Species distribution of the top BLAST hits for each unigene in Nr database. (**b**) Similarity distribution of the top BLAST hits for each unigene. (**c**) E-value distribution of BLAST hits for each unigene with a E-value cut off of 1.0E^−5^.

The highest percentage of blast hits came from *A*. *pisum* (85.3%). The species distribution of unigenes that have significant BLASTn hits against the NCBI nr protein database are shown in Fig. 3.

### Gene Ontology (GO) and Eukaryotic Othologous Groups classification (KOG)

The unigenes from *S*. *avenae* salivary glands were annotated for function using Gene Ontology (GO)^38^. Of the 33,079 assembled unigenes, 10,776 were assigned into 55 different functional groups. The three most abundantly represented categories in “biological process” were “cellular process”, “metabolic process” and “single-organism process” with 5,865 (54.43%), 5,232 (48.55%) and 4,415(40.97%) unigenes, respectively. In “cellular components” ontology, “cell” with 3,403 (31.58%) and “cell parts” with 3,403 (31.58%) unigenes were the two most common categories. In addition, in the “molecular function” group, unigenes were mainly distributed in two categories: “binding” with 6,108 (56.68%) unigenes and “catalytic activity” with 4,171 (38.70%) unigenes (Supplementary Figure 1).

EuKaryotic Orthologous Groups (KOG) is a version of the Clusters of Orthologous Groups (COG) for identifying orthologous and paralogous proteins in eukaryotic organisms^39^. In total, 7,956 unigenes were categorized into 26 groups, among these categories, the cluster of “General function prediction only” group had the most unigenes (1,425, 17.89%) followed by “Signal transduction mechanisms” (1,186, 14.91%), “Posttranslational modification, protein turnover, chaperones” (836, 10.51%), “Translation, ribosomal structure and biogenesis” (669, 8.41%), and “Transcription” (471, 5.92%) (Supplementary Figure 1).

### Metabolic pathway analysis by Kyoto Encyclopedia of Genes and Genomes (KEGG)

**Figure 4:**
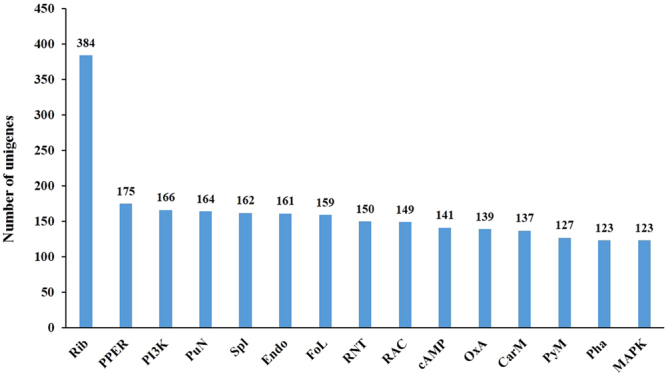
The top15 KEGG pathways with highest numbers of unigenes in salivary glands of *S*. *avenae*. Abbreviation for pathways: ribosome(Rib), protein processing in endoplasmic reticulum (PPER), PI3K-Akt signaling pathway (PI3K), purine metabolism (PuN), spliceosome (Spl), endocytosis (Endo), focal adhesion (FoL), RNA transport (RNT), regulation of actin cytoskeleton (RAC), cAMP signaling pathway (cAMP), oxidative phosphorylation (OxA), carbon metabolism (CarM), pyrimidine metabolism (PyM), phagosome (Pha), mitogen-activated protein kinase signaling pathway(MAPK).

When the Kyoto Encyclopedia of Genes and Genomes (KEGG) pathway was used to describe the network of molecular interactions and metabolic pathways in cells^40^, 6,772 unigenes in the salivary glands of *S*. *avenea* were mapped to a total of 229 KEGG pathways. Among these pathways, “Ribosome” (384 unigenes), “Protein processing in endoplasmic reticulum” (175 unigenes), and “PI3K-Akt signaling pathway” (166 unigenes) had the most unigenes (Fig. 4).

In the KO and KEGG annotations, the unigenes in the second hierarchy of the KEGG pathway were assigned to 5 categories, including “Cellular Processes”, “Environmental Information Processing”, “Genetic Information Processing”, “Metabolism” and “Organismal Systems” (Supplementary Figure 2). In the “Cellular Processes” category, “Transport and catabolism” and “Cellular community” were the two most common pathways with 463 (6.84%) and 344 (5.08%) unigenes, respectively. In the “Environmental Information Processing” category, “Signal transduction” included the most unigenes (935, 13.81%). In “Genetic Information Processing”, “Translation” and “Folding, sorting and degradation” were the top two pathways with 713 (10.53%) and 434 (6.41%) unigenes, respectively. In the “Metabolism” group, “Carbohydrate metabolism”, “Lipid metabolism” and “Amino acid metabolism” were the three most abundantly represented pathways with 346 (5.11%), 293 (4.33%) and 249 (3.68%) unigenes, respectively. In “Organismal Systems”, the unigenes were mainly assigned to “Endocrine system” (501, 7.40%), “Immune system” (355, 5.24%) and “Digestive system” (309, 4.56%). The KEGG pathway distributions were consistent with the characterization and potential function of aphid salivary proteins.

### Putative secretory proteins

Salivary proteins are proposed to be involved in interactions with plant, only when they can be secreted into plant during aphid probing and feeding. Therefore, all unigenes were analyzed for the presence of signal peptide and potential cleavage site using SignaIP software in this study. In total, 526 putative secretory proteins were obtained from all unigenes, however, some putative secretory proteins might have been missed as a result of partial ORF sequences, such as 3′ partial, 5′ partial and internal partial sequences. Of all putative secretory proteins, 335 (63.69%) proteins were functionally annotated and 191(36.31%) showed no similarities with known-function proteins in the Nr database. And, some function-annotated putative secretory proteins are very closely related to several insect salivary proteins that have been proved to play important roles in insect–host interactions, such as digestive and detoxifying enzymes and effectors (Supplementary Table 2).

#### Digestive and detoxifying enzymes

During phloem feeding, aphids secrete salivary proteins with digestive enzyme activity that facilitate probing and feeding. The transcript of a cell wall degradation enzyme, beta-mannosidase (c15158_g1) was found in our study. Beta-mannosidase catalyzes the endo-wise hydrolysis of the backbone of mannan and heteromannans, including a major component of plant cell walls, hemicellulose polysaccharides^41^. This enzyme can help aphids penetrate the plant cells. Sugar-degrading enzymes and proteases such as maltase, beta-glucuronidase, serine protease, trypsin and cathepsin were also identified in the salivary glands of *S*. *avenae* in our study. Wheat phloem sap is high in sucrose and some amino acids, predominantly glutamic acid, aspartic acid and serine^42^. The presence of these secretory digestive enzymes from *S*. *avenae* could thus aid extra-orally digestion, consistent with the feeding style of *S*. *avenae*.

When plants are attacked by herbivorous insects, plant hormones including jasmonic acid (JA), salicylic acid (SA), ethylene (ET), abscisic acid (ABA), and gibberellic acid (GA), known to modulate defense responses, are induced^43^. Plants also produce toxic secondary metabolites and defensive proteins, such as 2,4-dihydroxy-7-methoxy-2H-1,4-benzoxazin-3(4 H)-one (DIMBOA), lectins, glucosinolates, protease inhibitors to limit herbivory^44–47^. However, insects possess many enzymes to degrade toxins and facilitate host adaptation to adverse conditions. Cytochrome oxidases, glutathione *S*-transferases and esterase have been suggested as important enzymes for resistance to plant secondary metabolites and insecticides in aphids and other insects^48,49^. We identified the transcripts of cytochrome oxidase (c1987_g1), glutathione *S*-transferase 1 (c14396_g2), esterase FE4 (c7566_g1) and esterase E4 (c12530_g1) in the salivary glands of *S*. *avenae*. Previously, transcripts of cytochrome P450 oxidases and GSTs were found in the salivary gland of *E*. *fabae* ^23^, *N*. *lugens* ^25^, *N*. *cincticeps* ^26^, *A*. *pisum* ^29^ and protein cytochrome P450 oxidases and GST were also detected from saliva of Russian wheat aphid *Diuraphis noxia* ^50^, vetch aphid *Megoura viciae* ^51^ and *M*. *euphorbiae* ^52^. We also found metalloproteases (c14542_g1, c26916_g1) in *S*. *avenae*, a kind of peptidases, which were also identified in the saliva of *A*. *pisum*, *M*. *viciae* and *M*. *persicae* before^51^. Although the function of metalloproteases in aphid–plant interactions is still unclear, they are predicted to be involved in detoxifying plant defense proteins.

Peroxidase (c28134_g1, c13443_g1, c12708_g1, c14634_g2, c14243_g1, c14995_g1), an oxidoreductase was detected here, and was reported previously in salivary glands of Hession fly *Mayetiola destructor* ^53^, *A*. *pisum* ^29^ and *M*. *viciae* ^51^. Reactive oxygen species (ROS), including singlet oxygen, superoxide and hydrogen peroxide are involved in signaling pathways to activate plant defense responses and resistance to aphid. For example, accumulation of hydrogen peroxide at aphid feeding sites indicated reactive oxygen species are involved in early signaling of *A*. *thaliana* after infestation by the cabbage aphid *Brevicoryne brassicae* ^54^. High levels of H_2_O_2_ accumulated in a resistant near-isogenic wheat line but not in a susceptible line after infestation by the *D*. *noxia* ^55^. A sudden rise in oxygen concentration in sieve elements might result in protein coagulation in cucurbit phloem sap^56^. Additionally, high H_2_O_2_ levels in plants could be toxic to aphids. Peroxidase, acting as antioxidant enzymes involved in H_2_O_2_ scavenge, suggest that peroxidase detected in aphid salivary glands may protect aphids from plants oxidative stress as a detoxifying enzymes and play important roles in suppressing ROS production and ROS-induced plant defense responses.

#### Effectors eliciting or suppressing plant defenses

We found beta-glucosidase (c10709_g1) in the salivary glands of *S*. *avenae*. Beta-glucosidase, mainly regarded as a digestive enzyme for molecules such as hemicellulose, was also detected in the salivary glands of the termite *Neotermes koshunensi*s^57^. However, it might also be an effector to activate plant defense responses. Lima bean *Phaseolus lunatus* treated with a solution of beta-glucosidase emitted more volatile compounds, which were similar to those emitted in response to red spotted spider mite *Tetranychus urticae* infestation^58^. Leaves treated with commercial beta-glucosidase released volatile blends similar to that of leaves infested with cabbage white butterfly *Pieris brassicae* ^59^. Also, the levels of plant defense signaling molecules salicylic acid, ethylene, and H_2_O_2_ in rice increased after the application of beta-glucosidase, and the defense signaling pathways induced by beta-glucosidase were similar to those activated by infestation with *N*. *lugens* ^60^.

Lipases (c11727_g1, c8374_g1) and some phospholipases (c13818_g1, c7165_g1) were detected in the transcriptome of *S*. *avenae* salivary glands in this study. Lipases also have been found in the salivary glands of some insects, including mosquito *Anopheles stephensi* ^61^, *M*. *destructor* ^62^, large milkweed bug *Oncopeltus fasciatus* ^63^ and *E*. *fabae* ^23^. The primary function of lipases is presumed to be the breakdown membrane lipids and thereby the membrane; thus, they were first predicted to act as digestive enzymes to facilitate penetration of the cell membrane. However, Schäfer *et al*. showed that lipases in grasshopper oral secretions induced accumulation of cyclopentenone 12-oxo-phytodienoic acid (OPDA), a precursor of JA biosynthesis in *A*. *thaliana*, and external application of lipase solution to wounded leaves also highly increased the levels of 13-hydroperoxy octadecatrienoic acid, OPDA, JA, and jasmonic acid-isoleucine, suggesting that lipases elicited plant defense responses^64^. Phospholipases hydrolyze phospholipids, which take part in lipid synthesis, lipid-derived signaling pathway and plant stress responses. It is assumed that phospholipases D (PLD) and its products, phosphatidic acid, are involved in plant signal transduction cascades and the lipid metabolic pathway to influence plant stress responses. Gene expression and enzyme activity of PLD increased rapidly after exposure to various stresses, such as mechanical wounding, frost and pathogen infestation^65–67^. Increases in the PLD transcripts and accumulation of PLD along the plasma membrane were observed after rice leaves were infected with the bacterial pathogen *Xanthomonas oryzae* pv. *oryzae* ^68^. Also, PLD participates in the production of plant defense response signaling molecules (ABA, ET, NADPH oxidase)^66^. Thus, lipases in *S*. *avenae* or lipase-derived molecules are likely to serve as effectors to induce downstream plant defense responses.

Salivary glucose oxidase (GOX) was the first effector identified in the saliva of herbivores to suppress plant defense. The caterpillar *H*. *zea* secretes GOX into its host *Nicotiana tabacum* to suppress nicotine production and the jasmonic acid defense signaling pathway^4^. GOX was found in the saliva of many other caterpillar species and in the aphid *M*. *persicae* ^28^, but not in other aphid species. We identified another highly expressed glucose-methanol-choline oxidoreductase, glucose dehydrogenase (GLD, c10172_g2, c10815_g1, c12301_g1), in the salivary glands of *S*. *avenae*. GLD is also found in the saliva and salivary glands of other aphid species^29,34,50,51^. We predicted that GLD functioned in a similar way to GOX and as a potential effector to suppress plant defense responses after aphid infestation.

C002 is an aphid-specific watery saliva protein and also a well-known effector protein related to aphid feeding behavior and subsequent survival and fecundity^69–71^. Knockdown of C002 transcript of *A*. *pisum* and *S*. *graminum* resulted in high mortality of aphids^19,69,71^. The reproduction rate of *M*. *persicae* increased after feeding on host plants that over-express MpC002 but decreased after feeding on plants producing double-strand RNA (dsRNA) against C002^20,70^. At the proteomic level, C002 protein has been identified in *M*. *persicae* and *A*. *pisum* saliva^19,28,72^. However, its underlying mechanism is still unknown. An ortholog of C002 (c12732_g1) was also found in the salivary glands of *S*. *avenae* in this study with very high abundance in the transcriptome (RPKM = 10,926.33).

Angiotensin converting enzyme-1 (ACE-1, c10308_g1) was found in the salivary glands of *S*. *avenae* in the present study. ACE is a zinc-metallopeptidase found on the endothelial, epithelial and neuronal tissues in mammals^73^. The function of ACEs were studied in various insects such as locust *Locusta migratoria* ^74^, cotton leafworm *Spodoptera littoralis* ^75^ and buffalo fly *Haematobia irritans exigua* ^76^. Three ACE genes, ACE1, ACE2 and ACE3, were identified in the genome of *A*. *pisum* ^77^, and the ACE proteins were also detected in the saliva of *A*. *pisum* ^29,72^. ACE1 and ACE2 are highly expressed in the salivary glands, and compared with aphid survival rate on artificial diets, the survival rate of aphids with simultaneous knockdown of ACE1 and ACE2 decreased significantly after aphids fed on plants. These results indicated that ACE1 and ACE2 can function as effectors to modulate plant physiological processes to benefit aphid infestation^77^.

Trehalose, a kind of glucosidase in many organisms ranging from bacteria, fungi to plants^78^. Although the level of trehalose is very low in plants, many studies have demonstrated that it plays a regulatory role in sugar metabolism, growth, development and stress responses of plants^79^. Overexpressing trehalose biosynthetic genes of microbial and plant origin or receive exogenous trehalose, stress tolerance increased in several plants, such as tobacco, potato and rice^80,81^. We identified trehalase (c11521_g1, c13987_g1, c14644_g1), which degrades trehalose, in the salivary glands of *S*. *avenae*, and it has also been found in salivary glands of *B*. *tabaci* and the saliva of *M*. *dirhodum* and *S*. *avenae* ^24,34^. Trehalase activity in *A*.*thaliana* increased before trehalose accumulated when infested with the trehalose-producing pathogen *Plasmodiophora brassicae*, suggesting that trehalase may be a part of the plant defense responses and prevent excess accumulation of trehalose in plant cells^82^. Aphid-secreted trehalase may also play a role in interrupting trehalose accumulation to repress plant defense responses. However, a study showed that exogenous trehalose suppressed the transcript levels of some genes encoding enzymes related to plant defense, wound response, or pathogenesis, such as peroxidase-2 (PRXR2), basic endochitinase (ChiB), endo-1,3-b-d-glucanase (BGL1), lipoxygenase-2 (LOX2), and a chitinase-like protein 1 (CTL1)^83^. The role of trehalase in plant defense response needs further study.

Among the putative secretory proteins, we found odorant binding proteins (OBPs, c5921_g1, c2376_g1) and chemosensory proteins (CSPs, c5314_g1, c8576_g1, c18419_g1, c2724_g1). Transcripts of OBPs and CSPs have also been found in salivary glands of *N*. *lugens*, *M*.*persicae*, *M*.*cerasi* and bird cherry-oat aphid *Rhopalosiphum padi* ^25,84^. Insect OBPs and CSPs involved in olfaction and gustation are thought to be crucial for insect behaviors such as locating food and ovipository sites, as well as intraspecific communication^85^, and are mainly specifically expressed in chemosensory organs such as antennae and mouthparts and predicted to function in chemoperception^86–88^. However, some insect OBPs and CSPs are found in other tissues such as legs, heads, bodies and salivary glands^86^ with functions in insect development^89^, leg regeneration^90^, immune responses, and even interactions with the host. For example, Dengue virus (DENV) infection increased transcripts expression of OBP10 and OBP22 in the salivary glands of *Aedes aegypti* and silencing of the OBP10 or OBP22 genes resulted in a low efficiency of mosquito blood-feeding^91^. OBPs were found in the salivary glands of *Anopheles gambiae* and predicted to be secreted into host cells to manipulate host physiology by scavenging host amines^92^. Also, CSPs were identified using mass spectrometry as the most abundant proteins in the mandibular glands of larvae of the butterfly *Vanessa gonerilla* and speculated to play important roles in detecting microorganisms on plant surfaces, recognizing host plants and communicating with conspecifics^93^. CSP4 in cotton bollworm *Helicoverpa armigera* and oriental tobacco budworm *Helicoverpa assulta* possess unique functions that act as surfactants to reduce water surface tension and, consequently, pressure during sucking^94^, suggesting CSPs are involved in insect feeding. Among the CSPs identified in *S*. *avenae*, unigene c18419_g1 had high similarity to Mp10 known to be an effector in *M*. *persicae*. Mp10, a chemosensory protein was detected in the heads, salivary glands and whole bodies of *M*. *persicae*. Functional assays showed that overexpression of Mp10 in *N*. *benthamiana* suppressed bacterial pathogen-associated molecular pattern (PAMP) flg22-induced defense responses, but induced chlorosis and local cell death in *N*. *benthamiana*, resulting in a decrease of *M*. *persicae* fecundity^20,22^. Thus, OBPs and CSPs secreted by salivary glands of *S*. *avenae* may play important roles in aphid-host interactions and warrant further functional study.

Among potential effectors that were detected, such as lipid-binding proteins apolipophorins (c26673_g1)^51^, we also identified transcript sequence c10120_g1 as an ortholog of potato aphid *M*. *euphorbiae* effector Me10. Overexpression of Me10 in the host plant *N*. *benthamiana* increased *M*. *persicae* fecundity, suggesting its ability to suppress plant defenses^21,52^. As we mentioned before, some putative secretory proteins with effector activity may be missed because of partial sequences. For example, the unigene c9478_g1 (5′ primer partial) is a likely homolog of a potential salivary effector Mp55 of *M*. *persicae*, *A*. *thaliana* expressing Mp55 increased aphid reproduction, and accumulated less 4-methoxyindol-3-ylmethylglucosinolate, callose and hydrogen peroxide in response to aphid infestation, suggesting a role of Mp55 in suppressing plant defenses^95^.

#### Calcium ion ($C{a}^{2+}$) binding proteins

We identified some potential Ca^2+^ binding proteins in the putative secretory proteins of *S*. *avenae* salivary glands, including regucalcin (c11006_g1), reticulocalbin-2 (c8802_g1) and calumenin (c10782_g1).

Calcium ions (Ca^2+^) constitute a ubiquitous intracellular second messenger in many plant signaling pathways including induction of defense responses^96,97^. Mechanical damage of phloem tubes can trigger the sieve plate occlusion to avoid the outflow of phloem sap because of the releasing of Ca^2+^ into the sieve element lumen^98^. But damage caused by aphid stylets penetration don’t lead to the phloem sieve cell plugging^18^. Will T. firstly reported that *M*. *viciae* saliva contained some Ca^2+^ binding proteins prevent Ca^2+^-dependent sieve occlusion in *Vicia faba* through inducing dispersed forisomes, a Ca^2+^-driven contractile protein that can cause reversible plugs in sieve element, to return to the non-plugging state, and secretion of watery saliva also seems to be a universal way for aphids to suppress sieve-plate occlusion^99,100^. Some Ca^2+^ binding proteins were also found in salivary glands and saliva of hemipterans using transcriptomic and proteomic technologies, such as regucalcin in *A*. *pisum* and *N*. *cincticeps* ^26,72^, the most highly expressed salivary glands gene calcium-binding protein SP84(NcSP84) in *N*. *cincticeps* ^26^. These indicated that Ca^2+^ binding proteins secretion to suppress plant defense may be a common strategy amongst phloem-feeding insects.

### RT-PCR and RT- qPCR analysis of gene expression in different aphid tissues

The expression of the top 15 most highly expressed and some of interesting unigenes in salivary glands, alimentary canal, whole body minus salivary glands of apterous aphids were detected using RT-PCR and RT-qPCR. The 15 highly expressed transcripts, c10120_g1, c13498_g1, c12732_g1 (C002), c9723_g1, c8332_g1, c13074_g1 (micronuclear linker histone polyprotein-like , MLH), c6007_g1, c12301_g1 (glucose dehydrogenase, GLD), c9335_g1, c29740_g1 (tetratricopeptide repeat protein 21B, TPR), c3096_g1, c12029_g2 (glucose dehydrogenase, GLD), c10537_g1, c14714_g1, c15064_g1 and 9 interesting transcripts, c15158_g1 (beta-mannosidase, β-MAN), c15071_g1 (maltase A1, MAL), c14396_g2 (glutathione-S-transferase 1, GST-1), c13818_g1 (phospholipase D, PLD), c14644_g1 (trehalase, TRE), c10308_g1 (angiotensin converting enzyme, ACE) c11006_g1 (regucalcin, RGN), c8802_g1 (reticulocalbin-2, RCN), c10782_g1 (calumenin, CALU), are shown in (Supplementary Table 3).

**Figure 5:**
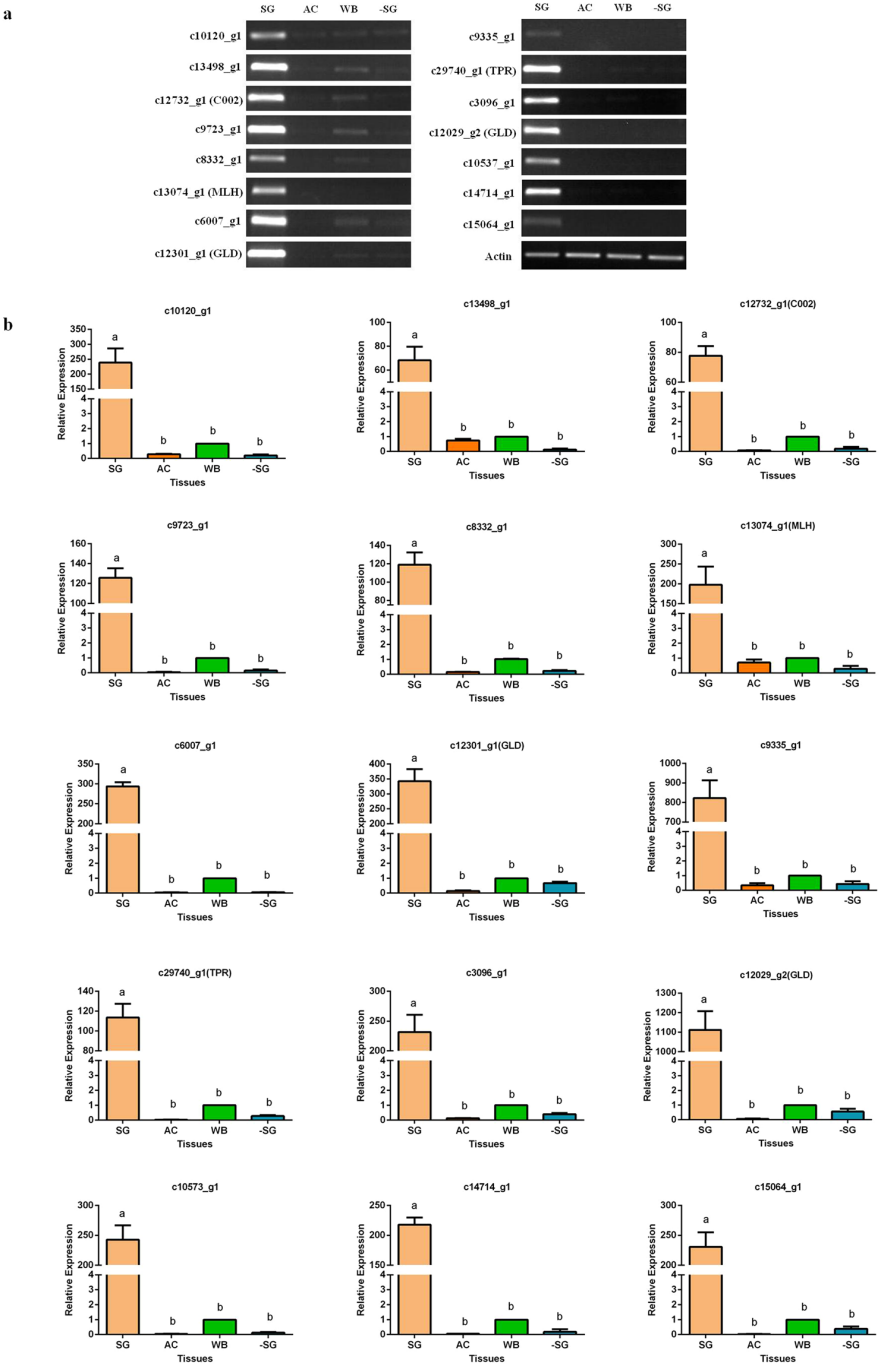
RT-PCR (**a**) and RT-qPCR (**b**) results of relative gene expression of the 15 most highly expressed putative secretory proteins of *S*. *avenae* salivary glands in different tissues. Abbreviation for unigenes: micronuclear linker histone polyprotein (MLH), glucose dehydrogenase (GLD); tetratricopeptide repeat protein (TPR). Tissues: salivary glands (SG); alimentary canals (AC); whole body of apterous adult (WB); whole body minus salivary glands of apterous adult (-SG). β-actin and NADH were used as an internal reference genes. In RT-PCR, the similar intensity of β-actin bands among different tissues indicates the equal concentrations of each template. Electrophoresis of all unigenes are run under the same PCR conditions. The display for each unigenes are cropped figures from the gels. The full-length gels are presented in Supplementary Figure 3. Standard error (SE) is represented by the error bar. Different small letters above each bar indicate significant differences (*P* < 0.05).

Interestingly, only 4 transcripts were annotated in top 15 highly expressed unigenes, with most still categorized as function unknown. Among these 4 annotated unigenes, GLD (c12301_g1, c12029_g2) was described before and predicted to be an aphid effectors to promote aphid infestation. Unigene c13074_g1 and c29740_g1 were annotated as   MLH and TPR, respectively. MLH is a DNA binding protein and involved in plant salt tolerance mechanisms. The gene expression for MLH polyproteins is upregulated in the salt-tolerant genotype of *Acer palmatum*, suggesting it is involved in the improvement of plant resistance to abiotic stress^101^. The TPR motif is a protein-protein interaction module, which is important for the functioning of chaperones, cell-cycle, transcription, protein transport complexes and the gibberellin signal transduction pathway^102,103^. Gibberellins (GAs) represent an important class of plant hormones that control growth and developmental processes. We predict that TPR secreted by aphids may have roles in the interactions with plant proteins and affect plant development. The RT-PCR and RT-qPCR results showed that all of these highly expressed transcripts were specifically expressed in aphid salivary glands (Fig. 5a,b). Relative expression of the most highly expressed unigenes c10120_g1 and c12029_g2 (GLD) in salivary gland was 238.5 ± 27.7 and 823.3 ± 51.9 times higher than in the whole body, respectively. These highly expressed salivary gland-specific genes may play vital roles in aphid-plant interactions, and are worth further analysis.

**Figure 6:**
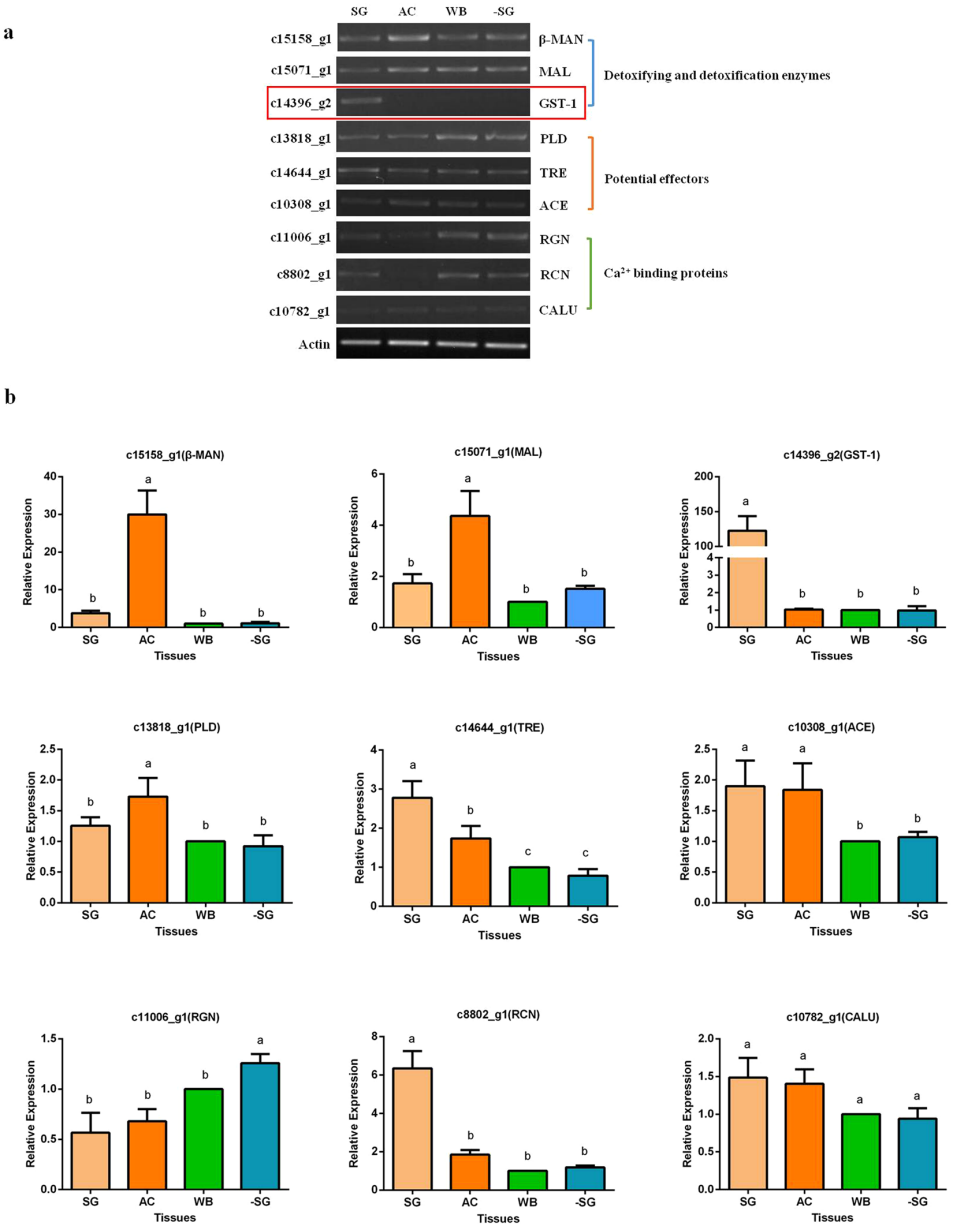
RT-PCR (**a**) and qRT-PCR (**b**) results of relative gene expression of 9 interested putative secretory proteins of *S*. *avenae* salivary glands in different tissues. The display for each unigenes are cropped figures from the gels. The full-length gels are presented in Supplementary Figure 4. Abbreviation for unigenes: beta-mannosidase (β-MAN), maltase A1 (MAL), glutathione-*S*-transferase 1 (GST-1) phospholipase D (PLD), trehalase (TRE), angiotensin-converting enzyme (ACE), regucalcin (RGN), reticulocalbin-2 (RCN), calumenin (CALU). Tissues: salivary glands (SG); alimentary canals (AC); whole body of apterous adult (WB); whole body minus salivary glands of apterous adult (-SG). β- actin and NADH were used as the internal reference genes. Standard error (SE) is represented by the error bar. Different small letters above each bar indicate significant differences (*P* < 0.05).

Among 9 genes of interest, β-MAN, MAL and PLD were detected in the transcriptome of *S*. *avenae* salivary glands, but the RT-PCR and RT-qPCR results showed that the expression levels of these transcripts in alimentary canal were significantly higher than in other tissues. The expression levels of ACE and putative Ca^2+^ binding proteins RCN in salivary glands were significantly higher than those in other tissues. GST-1 transcript was specifically expressed in the salivary glands. Aphid salivary protein with GST activity involved in modifying plant defense responses, overexpression of a single putative GST protein named Me47 detected in *M*. *euphorbiae* saliva could induce or suppress plant defense depending on different host plants^104^. TRE and CALU had high expression in both salivary glands and alimentary canal. In our study, RT-PCR and qPCR results showed that RGN was very low in the salivary glands compared with its levels in the alimentary canal and whole body (Fig. 6a,b). RGN was found exclusively in the anterior fat bodies of the flesh fly *Sarcophaga peregrine* ^105^, whereas the RGN protein was detected in the watery saliva of *A*.*pisum* ^34^, suggesting that RGN may be mainly produced in the alimentary canal and other tissues such as fat bodies, then transported from the haemolymph into the salivary gland to be secreted into the host.

**Figure 7:**
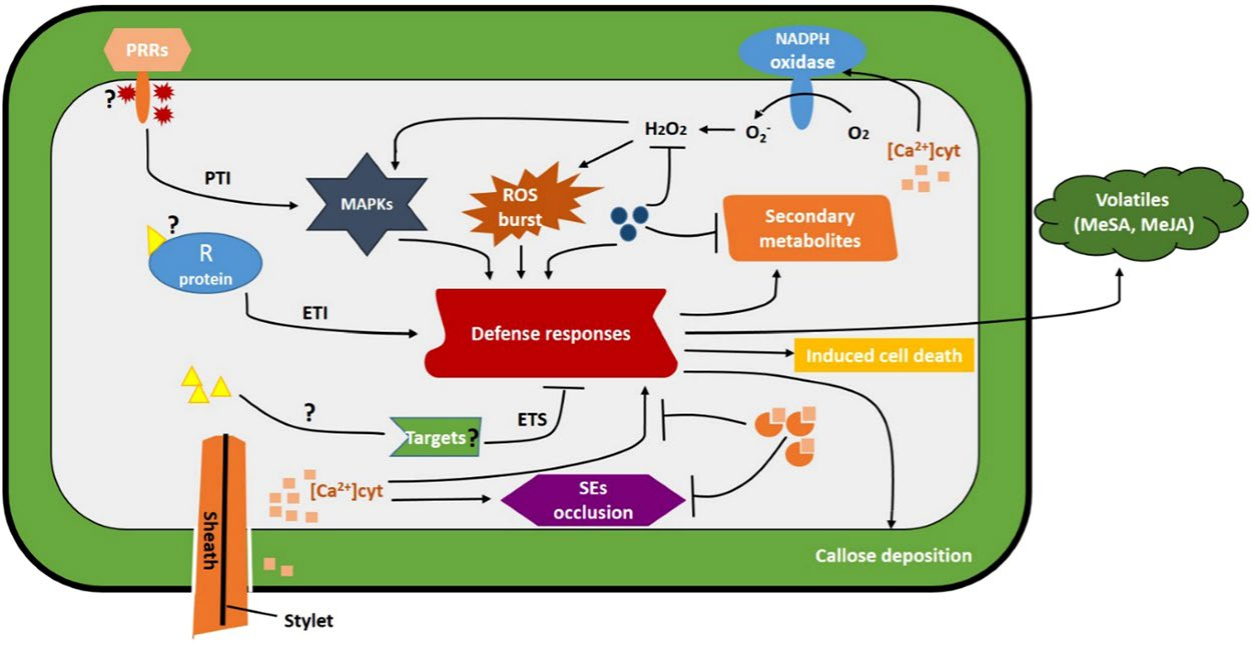
Schematic drawing of potential roles of secretory proteins from aphid saliva in aphid–plant interactions. During probing and feeding, the aphid secretes saliva into the cytoplasm of the plant cell. Digestive and detoxification enzymes () in salivary proteins facilitate probing and feeding because these enzymes are involved in the breaking down cell walls and membranes, metabolizing sugars and amino acids, and detoxifying secondary metabolites in plant tissues. Some digestive enzymes and degradative products also can induce plant defense responses as potential effectors. Some effectors () in saliva can be recognized by plant transmembrane pattern recognition receptors (PRRs), resulting in the induction of plant basal defenses (pattern-triggered immunity, PTI). Aphid also secretes other effectors () to suppress plant defenses to promote infestation, resulting in effector-triggered susceptibility (ETS). However, in resistant interactions, an effector () can be specifically recognized by plant resistance proteins (R proteins) according to the gene for gene hypothesis, resulting in a stronger defense responses, effector-triggered immunity (ETI) such as induced cell death. Penetration of plant cell membranes by aphids causes an increase in the cytosolic concentration of Ca^2+^ ([Ca^2+^]_cyt_), resulting in sieve pore occlusion and calcium signaling for defense responses such as the reactive oxygen species (ROS) burst. Ca^2+^ -binding proteins () injected with saliva can bind Ca^2+^, thereby preventing sieve pore blockage and calcium signaling pathways. Also, the hole in the membrane created by the aphid can be sealed after the secreted gelling saliva solidifies into a sheath, minimizing the influx of Ca^2+^ and downstream plant defense responses. However, few effectors have been identified, and details of the mechanisms involved in aphid–plant interaction are still unknown. Abbreviations: SEs: sieve elements MAPK: mitogen-activated protein kinase; MeSA: menthyl salicylate; MeJA: methyl jasmonate.

In conclusion, we revealed the transcripts of the salivary glands of *S*. *avenae* using Illumina HiSeq 2500. Five hundred and twenty five putative secretory proteins that were expected to be secreted into plants and may play critical roles in aphid-host interactions were identified. Among of them, some highly and salivary gland-specifically expressed genes were also uncovered, but most of these putative secretory proteins were still function-unknown, which are worthy of further study. We also suggest a model describing the potential roles of some salivary proteins in aphid-host interactions (Fig. 7). Further investigations are needed to confirm these predicted secreted proteins at the proteomic level for a more comprehensive understanding of the composition of its saliva. RNA interference (RNAi)^69–71^ and transient over-expression of aphid candidate effectors in model plants or non-model crops such as wheat or barley^20,106,107^ and yeast two-hybrid screening^108^ are also valuable approach to further investigate the function of aphid salivary proteins in aphid-host interactions.

## Materials and Methods

### Insect rearing

A clone of *S*. *avenae* was initially established from a single aphid collected from wheat field in Langfang, Hebei Province, China and has been reared on wheat plants (variety Beijing 837, which is susceptible to *S. avenae*) for 5 yr in an indoor environment at 20 ± 1 °C, 75–80% relative humidity and 16 h light/8 h dark.

### Sample collection and RNA isolation

About 600 pairs of salivary glands were individually dissected from apterous and alate adult aphids in phosphate buffered saline (pH = 7.2, Hyclone, Thermo Scientific, MA, USA) respectively, then quickly transferred to TRIzol Reagent (Invitrogen, Carlsbad, CA, USA) on ice. Total RNA was extracted using TRIzol Reagent following the manufacturer’s instructions and stored at −80 °C until used. RNA concentration was measured using Qubit RNA Assay Kit and Qubit 2.0 Flurometer (Life Technologies, CA, USA). RNA integrity was assessed using the RNA Nano 6000 Assay Kit and the Bioanalyzer 2100 system (Agilent Technologies, CA, USA). Three micrograms total RNA sample with standard quality (1.8 < OD260/280 < 2.1, RIN values > 8.0) was prepared for further sequencing.

### Illumina sequencing, assembly, and annotation

NEBNext^®^ Ultra™ RNA Library Prep Kit for Illumina^®^ (New England Biolabs (NEB), Beverly, MA, USA) was used to generate the sequencing libraries following the manufacturers’ introductions. Firstly, mRNA was purified from total RNA sample using poly-T oligo-attached magnetic beads. Fragmentation was performed using divalent cations under elevated temperature in NEBNext First Strand Synthesis Reaction Buffer (5×). First strand cDNA synthesis was carried out using M-MuLV Reverse Transcriptase and random hexamers, then second strand cDNA was synthesized using DNA polymerase I and RNase H. The remaining overhangs were converted into blunt ends using exonuclease/polymerase activities. After adenylation of 3′ ends of DNA fragments, NEBNext Adaptors were ligated to DNA fragments for hybridization. The library fragments were purified with the AMPure XP system to select the cDNA fragments with a length between 150 and 200 bp. Three microlitres USER Enzyme (NEB, USA) was used with size-selected, adaptor-ligated DNA at 37 °C for 15 min followed by 5 min at 95 °C, PCR was then performed to amplify cDNA with Phusion High-Fidelity DNA polymerase, Universal PCR primers and Index (X) Primer. The PCR products were purified with AMPure XP system and quantified using the Agilent Bioanalyzer 2100 system (Agilent Technologies, CA, USA). The clustering of the index-coded samples was performed on a cBot Cluster Generation System using TruSeq PE Cluster Kit v3-cBot-HS (Illumina, China) according to the manufacturer’s instructions.

After cluster generation, the library preparations were sequenced using an Illumina HiSeq. 2500/Miseq platform and paired-end reads (the sequencing strategy was PE150). The clean reads were obtained after adaptor sequences, ambiguous “N” nucleotides (the percentage of “N” > 10%) and low-quality sequences (the ratio of nucleotides with Qphred ≤ 5 was more than 50%) were removed from raw reads. The clean reads were assembled using Trinity r20140413p1 min_kmer_cov:2 and the other default parameters as described for de novo transcriptome assembly without a reference genome to generate transcripts and unigenes^109^. For homology searches and annotation, all unigenes were used in a search of public databases including non-redundant protein (Nr, e-value ≦ 1.0e^−5^), nucleotide sequence (Nt, e-value ≦ 1.0e^−5^), Pfam (e-value ≦ 0.01), euKaryotic Ortholog Groups (KOG)/Clusters of Orthologous Groups of proteins (COG, e-value ≦ 1.0e^−3^) and Swiss-Prot (e-value ≦ 1.0e^−5^). Functional annotation by Gene Ontology (GO) term was analyzed using Blast2GO with a cutoff value of E-value ≦ 1.0E^−6^. Pathways were annotated by KEGG Automatic Annotation Server (KAAS) based on the Kyoto Encyclopedia of Genes and Genomes (KEGG) with a cutoff value of E-value ≦ 1.0E^−10^.

### Putative secretory proteins

Open reading frames (ORFs) within transcript sequences generated by *de novo* RNA-Seq transcript assembly using Trinity were identified using TransDecoder v3.0.0 (https://github.com/TransDecoder/TransDecoder/releases). Signal peptides and cleavage sites in amino acid sequences were predicted by the SignaIP 4.1 Server (http://www.cbs.dtu.dk/services/SignalP/). For transmembrane domains prediction, amino acid sequences with a signal peptide were submitted to the TMHMM Server v. 2.0 (http://www.cbs.dtu.dk/ services/TMHMM/). Putative proteins with a signal peptide and 0–1 transmembrane domain (the signal peptide can be a transmembrane domain) were considered to be potential secreted proteins^25^.

### RT-PCR and RT-qPCR

Total RNA was extracted from 600 salivary glands, 450 alimentary canals, 10 whole bodies of apterous adult aphids and 10 whole bodies minus salivary glands as mentioned before. The cDNA was synthesized from 1 μg RNA using TransScript One-Step gDNA Removal and cDNA Synthesis SuperMix (TransGen Biotech, Beijing, China) for RT-PCR and qRT-PCR. All specific primers for RT-PCR and qRT-PCR were designed with Primer Premier 5.0 (PREMIER Biosoft, CA, USA) and are shown in (Supplementary Table 4). β-Actin and NADH dehydrogenase (NADH) were used as reference genes to normalize target gene expression^110,111^. All PCR products were sequenced (Sunbiotech, Beijing, China).

RT-PCR was conducted in a 20 μL reaction volume containing 10 μL 2 × Taq PCR MasterMix (BioMed, Beijing, China), 2 μL cDNA, 1 μL each forward and reverse primer (10 μM), 6 μL ddH_2_O. The RT-PCR conditions were 3 min at 94 °C; 28 cycles of 30 s at 94 °C, 30 s at 60 °C and 30 s at 72 °C; and final 10 min at 72 °C. PCR products were separated in 1% agarose gels and stained with ethidium bromide (Thermo Scientific, MA, USA). RT-qPCR was performed using an ABI 7500 Real-Time PCR System (Applied Biosystems, CA, USA).cDNA was diluted 10-fold and then used as the template to determine the relative expression of the target gene in a 20 μL reaction volume containing 2 μL cDNA, 0.5 μL each of 10 μmol L^−1^ forward primer and reverse primer, 10 μL of 2 × SYBR premix Ex Taq (Tli RNaseH Plus, Takara, Dalian, China) and 0.4 μL of 50 × ROX Reference Dye II (Tli RNaseH Plus, Takara, Dalian, China) at the following conditions: 30 s at 95 °C; followed by 40 cycles of 30 s at 95 °C, and 30 s at 60 °C. RT-qPCR reaction for each sample was carried out with 3 biological replicates and 3 technical replicates.

### RT-qPCR data analysis

Each treatment was performed in triplicate, and the differential expression was calculated using the 2^−ΔΔCT^ method^112^. The fold-change in expression of the unigenes in different tissues to the whole body of apterous adult aphid was calculated. Results were expressed as Means ± SE. All data were analyzed using SAS 9.1 software (SAS Institute Inc., NC, USA) and the differences among groups were examined using one-way analysis of variance (ANOVA) test. *P* values less than 0.05 were considered as statistically significant.

## Electronic supplementary material

Supporting information

Dataset2

Dataset1

Dataset3

Dataset4
