# Author Correction: Transcriptome analysis of the salivary glands of the grain aphid, *Sitobion avenae*

**DOI:** 10.1038/s41598-020-68520-2

**Published:** 2020-07-07

**Authors:** Yong Zhang, Jia Fan, Jingrui Sun, Frédéric Francis, Julian Chen

**Affiliations:** 1 State Key Laboratory of Plant Diseases and Insect Pests, Institute of Plant Protection, Chinese Academy of Agricultural Sciences, Beijing, 100193 People’s Republic of China; 2 Functional and Evolutionary Entomology, Gembloux Agro-Bio Tech, University of Liège, Gembloux, 5030 Belgium

Correction to: *Scientific Reports* https://doi.org/10.1038/s41598-017-16092-z, published online 21 November 2017

The information in this Article is incomplete. Since the publication of this Article, the SRA data has been at NCBI, under the accession number SRR11476040.

