# Supplementary material for: Transcriptome analysis of the salivary glands of the grain aphid, Sitobion avenae
Source: Sci Rep. 2017 Nov 21;7:15911. doi: 10.1038/s41598-017-16092-z (PMC5698471; doi:10.1038/s41598-017-16092-z)
Supplement: Supplementary file 1 — Supporting information [file 41598_2017_16092_MOESM1_ESM.pdf]

Transcriptome analysis of the salivary glands of the grain aphid, *Sitobion avenae*

Yong Zhang [1,2], Jia Fan [1], Jingrui Sun [1], Frédéric Francis[2*], Julian Chen [1*]

1 State Key Laboratory of Plant Diseases and Insect Pests, Institute of Plant Protection, Chinese Academy of Agricultural Sciences, Beijing, 100193, PR China

2 Functional and Evolutionary Entomology, Gembloux Agro-Bio Tech, University of Liège, Gembloux, B-5030, Belgium

Corresponding authors: Julian Chen, Tel: +86 10 62813685; E-mail: jlchen@ippcaas.cn ; Frédéric Francis, Tel.: +32 81 622283; E-mail: Frederic.Francis@ulg.ac.be.

Supplementary Figures

Supplementary Figure 1. The enriched GO and KOG molecular functions for all salivary gland-expressed genes.

Supplementary Figure 2. The KEGG pathway for all salivary gland-expressed genes.

Supplementary Figure 3. The full-length gels for each unigenes presented in Figure 5a.

Supplementary Figure 4. The full-length gels for each unigenes presented in Figure 6a.

Supplementary Tables

Supplementary Table 1. List of all unigenes in the salivary glands of *S.avenae*.

Supplementary Table 2. List of genes with a putative signal peptide.

Supplementary Table3. List of top 15 highly expressed genes and 9 interested genes in salivary glands of *S.avenae.*

Supplementary Table 4 List of all primers used in RT-PCR and qRT-PCR

Supplementary Figure 1. The enriched GO and KOG molecular functions for all salivary gland-expressed genes.

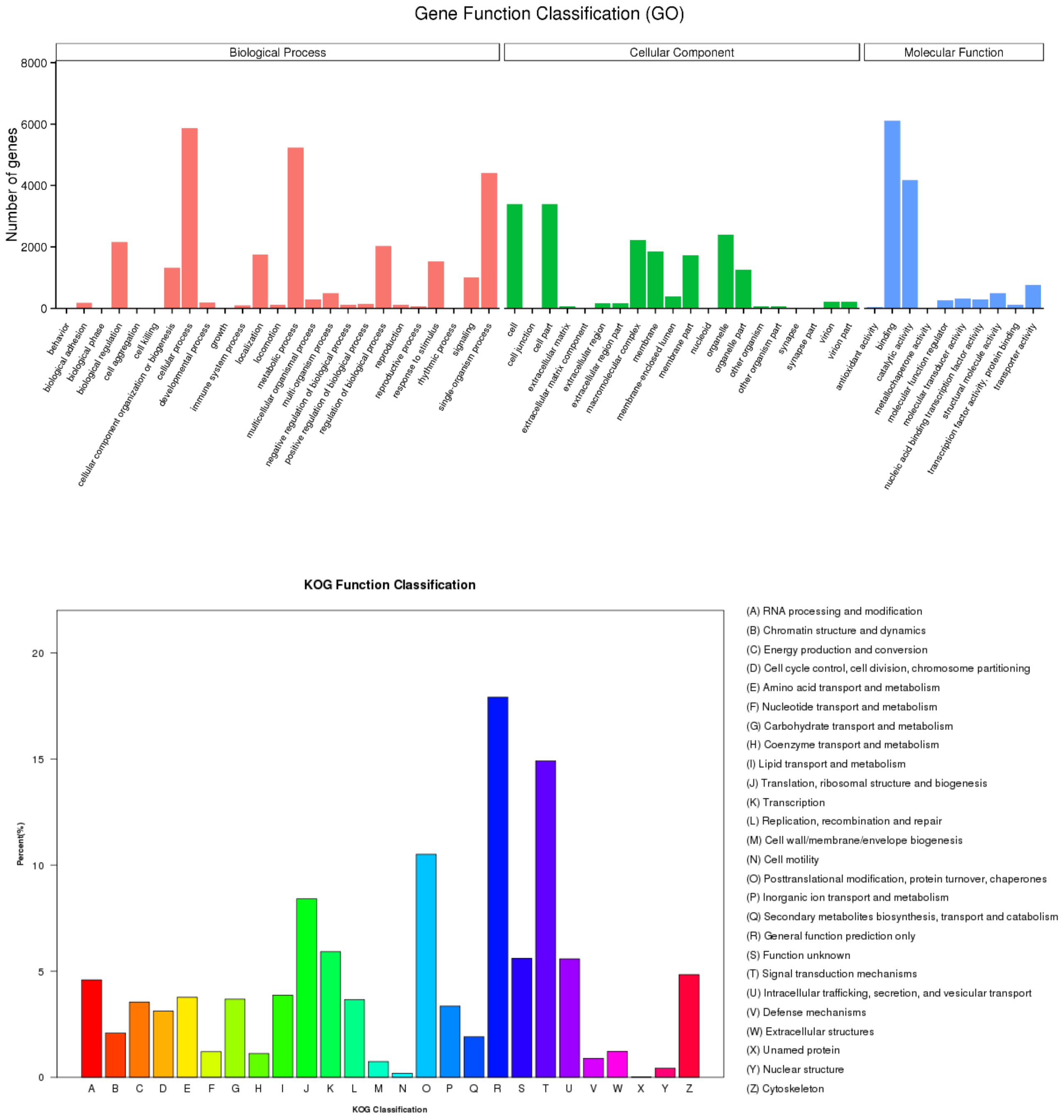

Supplementary Figure 2. The KEGG pathway for all salivary gland-expressed genes. A: Cellular Processes; B: Environmental Information Processing; C: Genetic Information Processing; D: Metabolism; E: Organismal Systems.

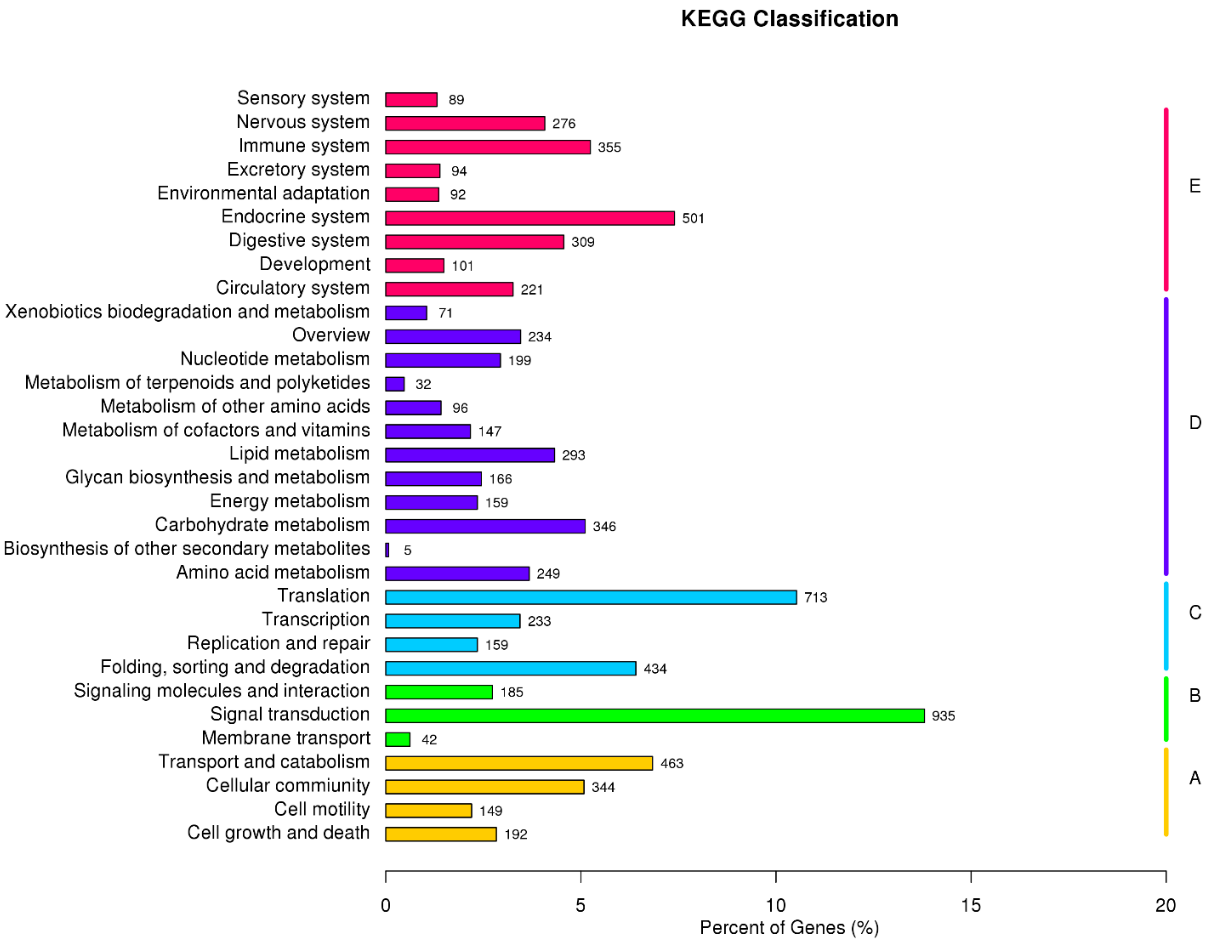

Supplementary Figure 3. The full-length gels for each unigenes presented in Figure 5a.

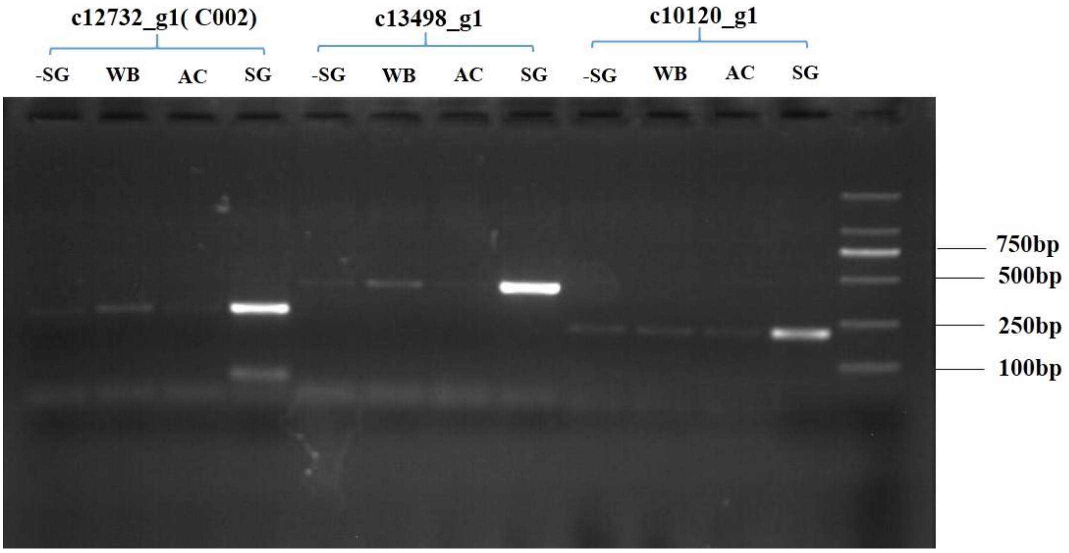

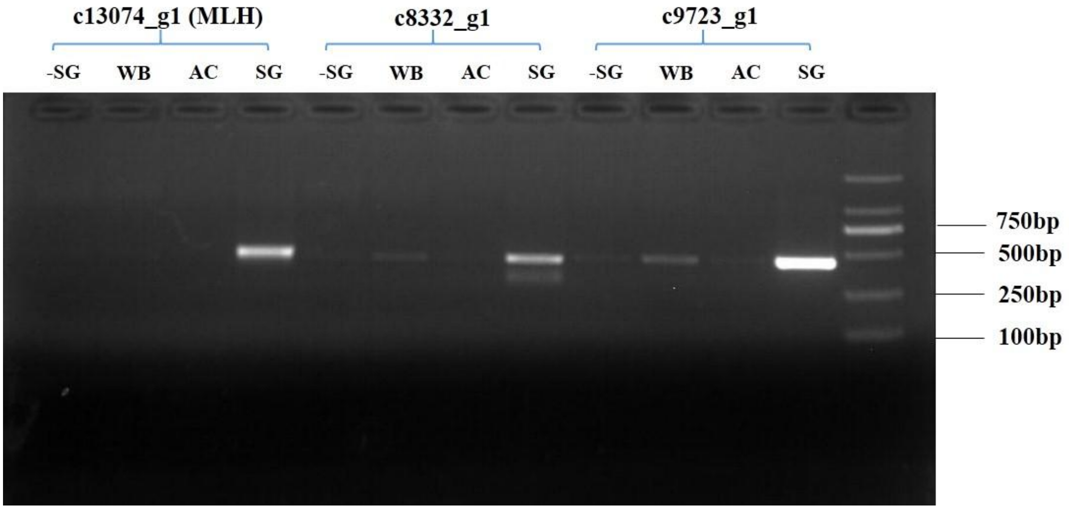

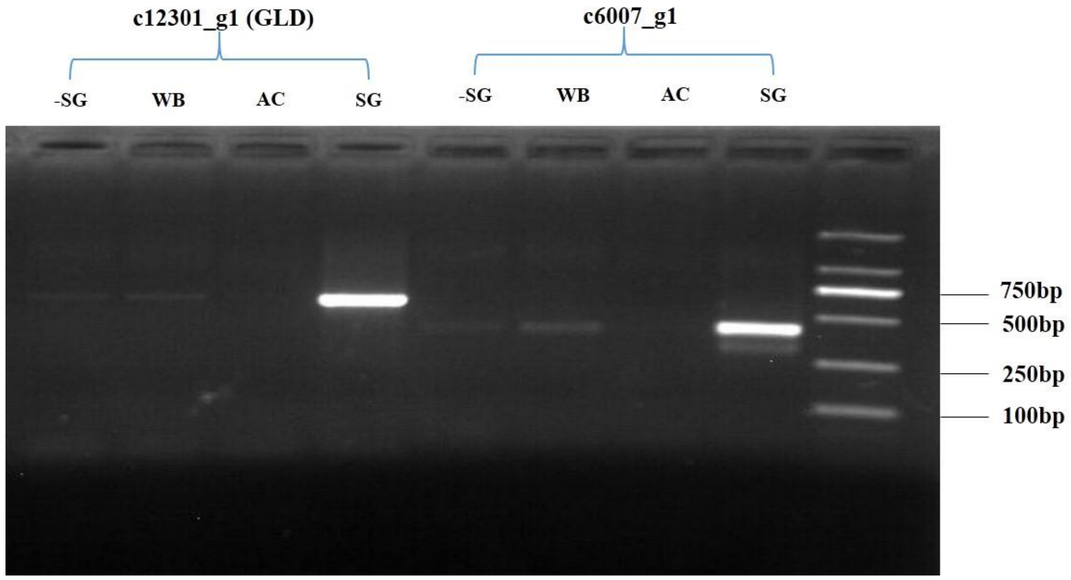
c12301_g1 (GLD)
c6007_g1
-SG
WB
AC
SG
-SG
WB
AC
SG
750bp
500bp
250bp
100bp

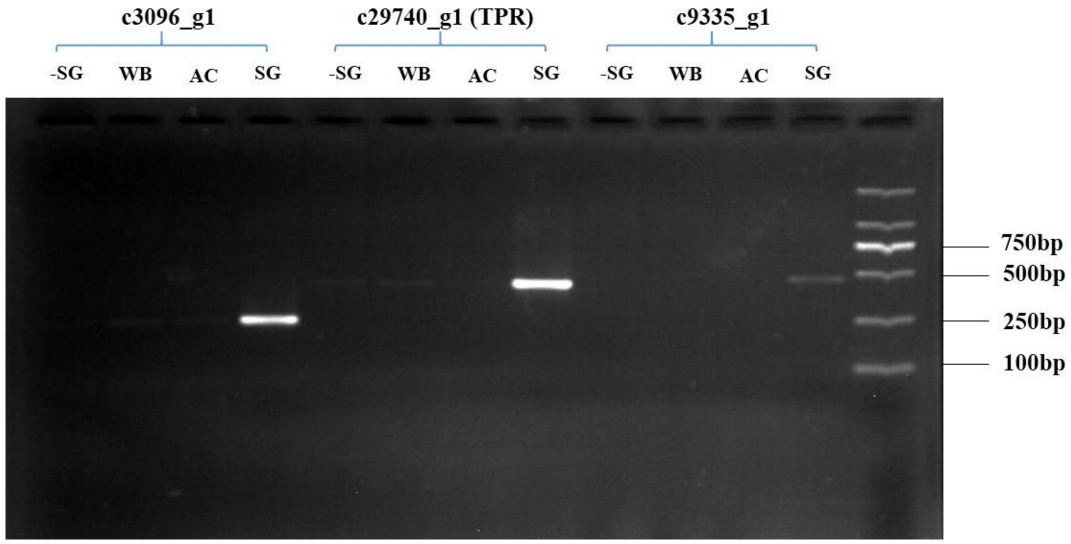
c3096_g1
c29740_g1 (TPR)
c9335_g1
-SG
WB
AC
SG
-SG
WB
AC
SG
-SG
WB
AC
SG
750bp
500bp
250bp
100bp

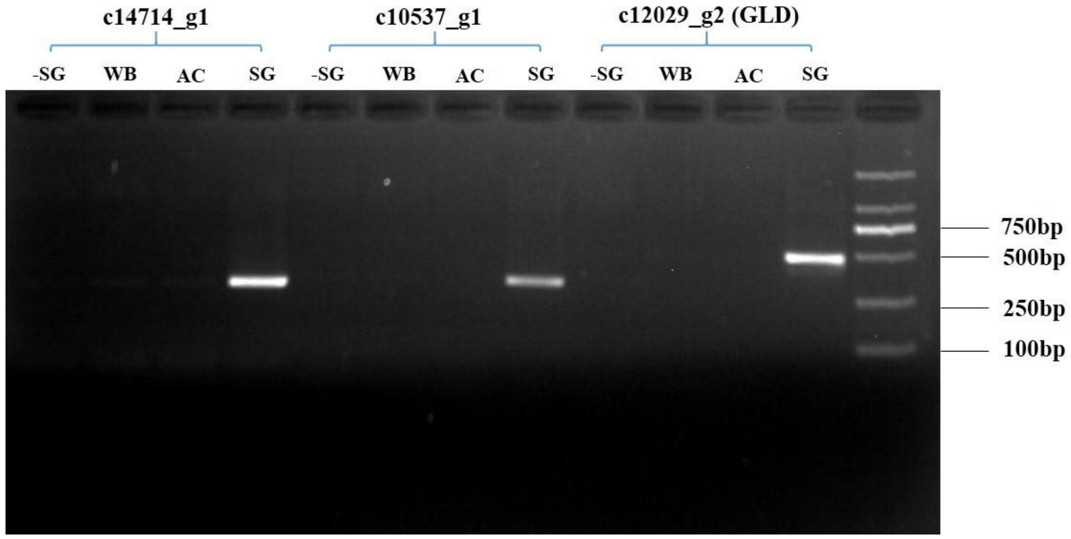
c14714_g1
c10537_g1
c12029_g2 (GLD)
-SG
WB
AC
SG
-SG
WB
AC
SG
-SG
WB
AC
SG
750bp
500bp
250bp
100bp

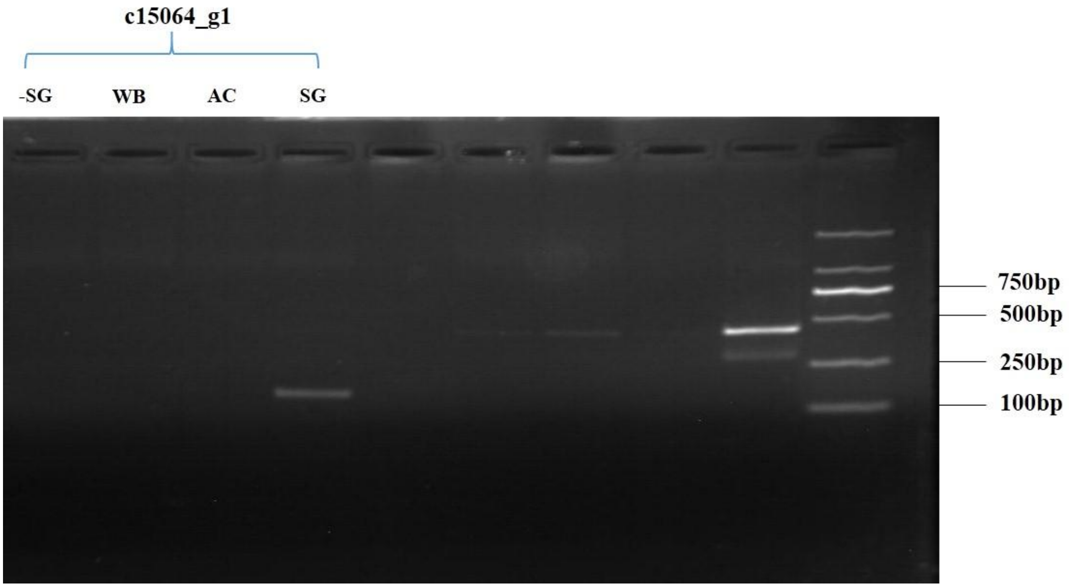

Supplementary Figure 4. The full-length gels for each unigenes presented in Figure 6a.

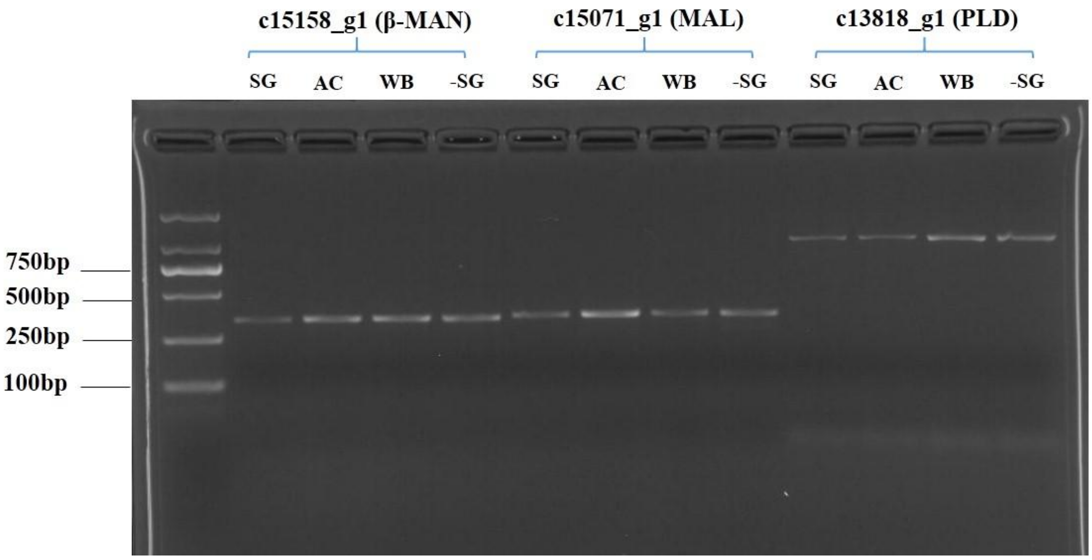

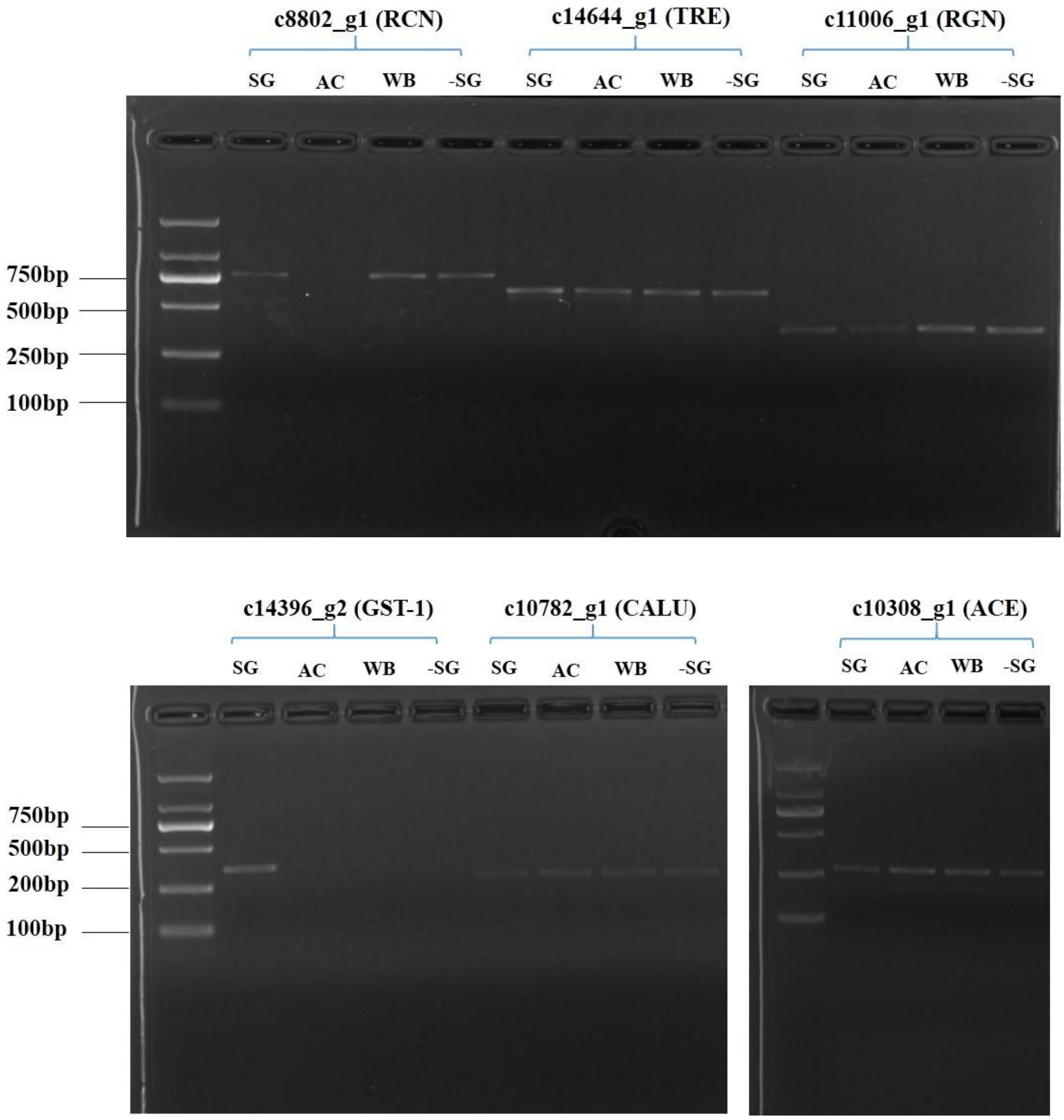
c8802_g1 (RCN)
c14644_g1 (TRE)
c11006_g1 (RGN)
SG AC WB -SG SG AC WB -SG SG AC WB -SG
750bp
500bp
250bp
100bp
c14396_g2 (GST-1)
c10782_g1 (CALU)
c10308_g1 (ACE)
SG AC WB -SG SG AC WB -SG SG AC WB -SG
750bp
500bp
200bp
100bp
